# A protein phosphatase 2C, AP2C1, interacts with and negatively regulates the function of CIPK9 under potassium-deficient conditions in Arabidopsis

**DOI:** 10.1093/jxb/ery182

**Published:** 2018-05-15

**Authors:** Amarjeet Singh, Akhilesh K Yadav, Kanwaljeet Kaur, Sibaji K Sanyal, Saroj K Jha, Joel L Fernandes, Pankhuri Sharma, Indu Tokas, Amita Pandey, Sheng Luan, Girdhar K Pandey

**Affiliations:** 1 Department of Plant Molecular Biology, University of Delhi South Campus, New Delhi, India; 2 Department of Plant and Microbial Biology, University of California Berkeley, Berkeley, California, USA

**Keywords:** Arabidopsis, calcium signaling, CBL-interacting protein kinase, dephosphorylation, phosphorylation, potassium deficiency, protein phosphatase 2C, regulation

## Abstract

Potassium (K^+^) is a major macronutrient required for plant growth. An adaptive mechanism to low-K^+^ conditions involves activation of the Ca^2+^ signaling network that consists of calcineurin B-like proteins (CBLs) and CBL-interacting kinases (CIPKs). The CBL-interacting protein kinase 9 (CIPK9) has previously been implicated in low-K^+^ responses in *Arabidopsis thaliana*. Here, we report a protein phosphatase 2C (PP2C), AP2C1, that interacts with CIPK9. Fluorescence resonance energy transfer (FRET), bimolecular fluorescence complementation (BiFC), and co-localization analyses revealed that CIPK9 and AP2C1 interact in the cytoplasm. AP2C1 dephosphorylates the auto-phosphorylated form of CIPK9 *in vitro*, presenting a regulatory mechanism for CIPK9 function. Furthermore, genetic and molecular analyses revealed that *ap2c1* null mutants (*ap2c1-1* and *ap2c1-2*) are tolerant to low-K^+^ conditions, retain higher K^+^ content, and show higher expression of K^+^-deficiency related genes contrary to *cipk9* mutants (*cipk9-1* and *cipk9-2*). In contrast, transgenic plants overexpressing AP2C1 were sensitive to low-K^+^ conditions. Thus, this study shows that AP2C1 and CIPK9 interact to regulate K^+^-deficiency responses in Arabidopsis. CIPK9 functions as positive regulator whereas AP2C1 acts as a negative regulator of Arabidopsis root growth and seedling development under low-K^+^ conditions.

## Teaser

AP2C1 dephosphorylates CIPK9 to negatively regulate its function in controlling root growth and seedling development under low-K^+^ conditions in Arabidopsis.

## Introduction

Potassium (K^+^) is the most abundant cation and an essential macronutrient in living plant cells. It constitutes almost 10% of the plant’s dry weight and is vital in many physiological processes in plant cells, including electrical neutralization, enzyme activation, stomatal movement, maintenance of membrane potential, and osmotic regulation (Han *et al.*, 2016). Moreover, K^+^ participates in accentuating photosynthesis, starch synthesis, and transport of assimilates, thereby ultimately determining crop yield and productivity (Pettigrew, 2008; Zörb *et al.*, 2014). In the cytoplasm, the K^+^ concentration remains stable at approximately 100 mM (Walker *et al.*, 1996); however, its concentration in the soil at the root surface is generally as low as 1 mM (Schroeder *et al.*, 1994; Maathuis, 2009).

Due to its requirement in several vital cellular processes, K^+^ deficiency adversely affects plant growth and development (Wang and Wu, 2013). In order to counteract and adapt to an environment deficient in K^+^, plants have evolved complex signaling and physiological regulatory systems (Wang and Wu, 2013). One of the major mechanisms is maintenance of cellular K^+^ homeostasis, which involves uptake and transport of K^+^ across different membranes (Amtmann and Armengaud, 2007). Extensive molecular analysis of the transport system has suggested two main mechanisms of K^+^ acquisition, namely high- and low-affinity uptake through roots. High-affinity uptake is carried out by transporters at low external K^+^ concentrations (as low as 10 μM), whereas low-affinity uptake and transport is mediated by K^+^ channels at relatively higher external concentrations (above 100 μM) (Rubio *et al.*, 2008; Pyo *et al.*, 2010; Shin, 2014).

In Arabidopsis under low-K^+^ conditions, a specific Ca^2+^ signal is generated through an unknown mechanism (Behera *et al.*, 2017) and transduced downstream by a module consisting of calcineurin B-like (CBL) protein and CBL-interacting kinase (CIPK) (Singh *et al.*, 2016). The Ca^2+^ signal is generally perceived by the Ca^2+^ sensors and their interactors/targets. The CBL proteins have been recognized as crucial Ca^2+^ sensors in plant cells (Pandey, 2008; Luan, 2009) that interact with CIPKs, a family of plant serine/threonine kinases (Pandey, 2008; Luan, 2009; Pandey *et al.*, 2014). A total of 10 CBL and 26 CIPK isoforms have been identified in the Arabidopsis genome. Different CBL-CIPK isoforms constitute diverse interacting networks to regulate Ca^2+^ signaling pathways (Luan, 2009; Weinl and Kudla, 2009; Pandey *et al.*, 2014). Under K^+^-deficient conditions, the CBL1/CBL9-CIPK23 complex localizes to the plasma membrane of root hair cells, where CIPK23 phosphorylates the voltage-dependent potassium channel AKT1 (Arabidopsis K^+^ transporter 1) to enhance K^+^ uptake (Li *et al.*, 2006; Xu *et al.*, 2006). Using a reverse genetic screen, CIPK9 was identified as an important regulator of the K^+^-deficiency response in Arabidopsis (Pandey *et al.*, 2007). Loss-of-function of CIPK9 leads to enhanced sensitivity on low-K^+^ media in terms of plant growth, suggesting that it functions as a positive regulator of low-K^+^ tolerance (Pandey *et al.*, 2007). In addition to CIPKs, calcium-dependent protein kinases (CDPKs) and sucrose non-fermenting-1-related protein kinase 2s (SnRK2s) have been found to regulate K^+^ channels that are involved in stomatal movement during drought stress. For example, Arabidopsis AtCPK13 in guard cells phosphorylates the inward-rectifying K^+^ channels AtKAT1 and AtKAT2. Similarly, AtSnRK2.6 (AtOST1) phosphorylates AtKAT1, leading to its inactivation and thereby promoting stomatal closure (Simeunovic *et al.*, 2016). Furthermore, AtCPK3, 4, 5, 11, and 29 phosphorylate the two-pore K^+^ channel AtTPK1, which is responsible for K^+^ efflux from the vacuole in Arabidopsis (Latz *et al.*, 2013; Simeunovic *et al.*, 2016).

Protein phosphatase 2Cs (PP2Cs) are known as counteracting molecules to kinases in general, and are also involved in K^+^ deficiency-triggered signaling (Chérel *et al.*, 2002; Lee *et al.*, 2007; Singh *et al.*, 2016). While the CBL1/CBL9-CIPK23 complex functions to activate AKT1 through phosphorylation, a member of the PP2C family, AIP1, deactivates AKT1 by dephosphorylation (Li *et al.*, 2006; Lee *et al.*, 2007). This CBL1/CBL9-CIPK23–AKT1–AIP1 module is the primary signaling cascade that has so far been identified to regulate K^+^ uptake in plants (Wang and Wu, 2017). AtPP2CA interacts with the Arabidopsis K^+^ transporter AKT2, which is a weak inward-rectifier channel. Co-expression of AtPP2CA and AKT2 in fibroblast-like COS cells and *Xenopus* oocytes results in inhibition of the AKT2 inward-rectification current: inhibition of the AKT2-mediated current is due to dephosphorylation of AKT2 by AtPP2CA (Chérel *et al.*, 2002) In addition, it was found that the activity of the AKT2 channel and its translocation from the endoplasmic reticulum to the plasma membrane was regulated through the interaction of the CBL4-CIPK6 complex, but this was independent of phosphorylation activity (Held *et al.*, 2011). To date, no kinases that can phosphorylate AKT2 have been identified at the molecular level (Wang and Wu, 2013), but it is clear that phosphorylation and dephosphorylation of K^+^ channels is an important regulatory mechanism of uptake and homeostasis under low-K^+^ conditions.

In this study, we identified a protein phosphatase 2C, AP2C1, as an interactor of CIPK9. The physical interaction of AP2C1 and CIPK9 was confirmed by yeast two-hybrid and protein pull-down assays. The *in planta* interaction of the two proteins was established by fluorescence resonance energy transfer (FRET), bimolecular fluorescence complementation (BiFC), and co-localization assays. Biochemical activity analyses showed that AP2C1 dephosphorylates auto-phosphorylated CIPK9, which could be the possible regulatory mechanism of CIPK9 function *in vivo*. Genetic and molecular analyses of AP2C1 and CIPK9 null mutants and AP2C1-overexpressing transgenic plants suggested the functional involvement of CIPK9 and AP2C1 in the same signaling pathway to regulate K^+^-deficiency responses in Arabidopsis.

## Materials and methods

### Preparation of constructs and yeast two-hybrid analysis

Complete amplified ORFs of *AP2C1* and *CIPK9* were inserted at the *BamH*I and *Sal*I restriction sites of both the activation-domain vector pGAD.GH and the DNA binding-domain vector pGBT9.BS (Clontech). Similarly, different *AP2C1* deletion fragments (K1, K2, K3, KIM, and PP2Cc) and other fragments were cloned into the *BamH*I and *Sal*I restriction sites of both the pGAD.GH and pGBT9 vectors. All the clones were confirmed by sequencing and primers are listed in Supplementary Table S1 at *JXB* online. To examine the physical interaction between AP2C1 and CIPK9, AD-AP2C1 and BD-CIPK9 (and also vector-swap) plasmids were co-transformed into yeast strain AH109 and yeast two-hybrid assays were performed according to Pandey *et al.* (2015). Similar analyses were also performed for AP2C1 with all the 26 members of the Arabidopsis CIPK family (including CIPK9), and for CIPK9 with PP2Cs that are homologs of AP2C1 and that have previously been implicated in the K^+^-deficiency response (Lee *et al.*, 2007; Lan *et al.*, 2011).

### Protein expression and purification

To express proteins in *E. coli*, complete ORFs lacking the stop codon of *AP2C1* and *CIPK9* were amplified and cloned in fusion with GST and 6X-His tags of the pGEX4T-1 (GE HealthCare, USA) and pET28a (Novagen) vectors, respectively, at the *BamH*I and *Sal*I restriction sites. All the clones were confirmed by sequencing and the primers are listed in Supplementary Table S1. Protein expression and purification were carried out according to Sanyal *et al.* (2017).

### Site-directed mutagenesis of AP2C1 (G178D)

The substitution of Gly^178^ (G) to Asp^178^ (D) in the AP2C1 ORF was carried out using a QuikChange Site-Directed Mutagenesis Kit (Stratagene) according to the manufacturer’s instructions, using following primer pair: forward, TTCGGAGTCTATGATGGTCAT*GAC*GGAGTTAAAGC GGCTGAGTTT; reverse, AAACTCAGCCGCTTTAACTCC*GTC* ATGACCATCATAGACTCCGAA. Mutagenesis reactions were conducted on AP2C1/pDONOR (ABRC, Ohio) plasmid DNA. A 50-μl PCR reaction was set up using 50 ng of template plasmid, 200 μM of dNTPs, 125 ng of primers, and 1 unit of PrimeSTAR™ DNA polymerase (Takara, Japan). The PCR conditions for the reaction were: 98 °C for 4 min, followed by 18 cycles of 98 °C for 30 s, 68 °C for 1 min, 72 °C for 6 min, followed by final extension at 72 °C for 10 min. The construct was verified for the mutagenesis by sequencing. The mutated AP2C1 (G178D) sequence was amplified and sub-cloned into pGEX4T-1 for protein expression.

### *In vitro* GST-pull down assay

Recombinant proteins with the GST tag were purified using Glutathione-sepharose 4B according to manufacturer’s instructions (GE-Healthcare, UK). The proteins expressing the 6X-His tag were induced using 0.1 mM isopropyl-1-thio-b-D-galactopyranoside (IPTG) at 30 °C for 6 h. The expressed soluble protein was extracted in 1× phosphate buffer saline (pH 7.5) with lysozyme and sonication. The purified protein was quantified by loading onto a 10% SDS-PAGE gel and staining with Coomassie brilliant blue. The Glutathione-sepharose 4B-bound GST and GST-fused proteins (approximately 1 μg) were incubated with bacterial soluble lysate expressing 6X-His fusion proteins for 4 h at 4 °C. The beads were washed six times with phosphate buffer saline at 4 °C. Protein complexes bound to the beads were boiled in 2× SDS sample buffer for 5 min and separated by 10% SDS-PAGE. Proteins were transferred to a nitrocellulose membrane. Immunoblotting was performed using an anti-His antibody (1:5000 dilution) and loading controls were performed with an anti-GST antibody (1:500 dilution).

### *In vitro* phosphatase activity assay

Purified GST-CIPK9, GST-AP2C1, and GST-AP2C1 (G178D) proteins were quantified with known concentrations of purified bovine serum albumin (BSA). *In vitro* assays were performed in reaction buffer (20 mM Tris pH 7.2, 2.5 mM MnCl_2_, 0.5 mM CaCl_2_, 1 mM DTT, 10 μM ATP, and 5 μCi ^32^γP) with different combinations of affinity-purified GST-CIPK9 (1 μg) and GST-AP2C1(500 ng) proteins in a 30-μl reaction. The reaction was performed at 30 °C for 30 min. and terminated by 1× SDS-PAGE loading buffer. The reactions were loaded onto 10% SDS-PAGE, and after resolution the gels were dried and signals were developed by exposing the dried gel to X-ray film using the autoradiography methodology. To determine the dose-dependent phosphatase activity of AP2C1, different concentrations of GST-AP2C1 and GST-AP2C1 (G178D) (10–250 ng) were used with a constant amount of GST-CIPK9 (500 ng).

### Fluorescence resonance energy transfer (FRET) analysis

The complete ORFs lacking the stop codon of *CIPK9* and *AP2C1* were amplified from cDNA, cloned in pENTR-D/TOPO (Invitrogen), and subsequently mobilized to the Gateway-compatible binary vectors pSITE 3CA (yellow fluorescent protein tag, YFP) and pSITE 1CA (cyan fluorescent protein tag, CFP), respectively (Chakrabarty *et al.*, 2007). Primers are listed in Supplementary Table S1. *Nicotiana benthamiana* plants were transiently transformed with *Agrobacterium tumefaciens* GV3101::pMP90 carrying the AP2C1-CFP, KIM-CFP, and K2-CFP constructs in combination with YFP-CIPK9 according to Singh *et al.* (2013). Transformed cells were analysed using a confocal microscope (TCS SP5; Leica). Cells showing expression of both CFP and YFP were selected for FRET analysis according to the acceptor-bleaching protocol. FRET efficiency was recorded in at least seven different cells. CFP was excited by an argon laser at 458 nm and emissions were detected between 465–505 nm. YFP was excited at 512 nm and emissions were detected between 525–600 nm. Empty vectors pSITE3CA and pSITE1CA were co-transformed as negative controls.

### Bimolecular fluorescence complementation (BiFC) and co-localization assays

The amplified ORF of *AP2C1* was inserted at the *BamH*I and *Sal*I restriction sites of the binary vector pGPTVII.GFP.Kan. The coding region of *CIPK9* was cloned in the Gateway entry vector pENTR-D/TOPO (Invitrogen) and subsequently mobilized to the Gateway-compatible binary vector pSITE4CA. Primers are listed in Supplementary Table S1. These constructs were used to transform *N. benthamiana* cells as describe above. For co-localization, cultures of *Agrobacterium* carrying AP2C1-GFP (green fluorescent protein) and RFP-CIPK9 (red fluorescent protein) were grown overnight, then mixed and infiltrated into *N. benthamiana* cells. For BiFC, ORFs of AP2C1 and CIPK9 were mobilized using Gateway technology from the pENTR-D/TOPO vector to pSPYCE-35S^GW^ and pSPYNE-35S^GW^, respectively. *Agrobacterium* GV3101::pMP90 carrying the BiFC constructs were co-infiltrated in *N. benthamiana* cells.

### Confocal microscopy

Transiently transformed *N. benthamiana* epidermal peel cells were analysed using a confocal microscope to detect the fluorescence. Confocal microscopy was performed according to Singh *et al.* (2014).

### Plant material and growth conditions

*Arabidopsis thaliana* ecotype Columbia-0 was used for generation of overexpression transgenic plants. Arabidopsis seeds were surface-sterilized and grown according to Singh *et al.* (2015).

### Isolation of T-DNA insertion mutants

The homozygous mutant allele *ap2c1-1* (SALK_065126) was kindly provided by Dr. Irute Meskiene (Max F. Perutz Laboratories, University of Vienna, Austria). The *ap2c1-2* mutant allele was isolated from the T-DNA insertion collection of the Arabidopsis Biological Resource Centre–SALK (http://signal.salk.edu/; SALK_104445). T-DNA insertion and its genomic position were confirmed by PCR using a T-DNA left border and a gene-specific primer. After the selfing of heterozygous plants, the homozygous *ap2c1-2* mutant allele was identified and disruption of the native AP2C1 gene was confirmed by genomic DNA PCR. Disruption of the gene expression in both alleles was confirmed by semi-quantitative RT-PCR. The homozygous CIPK9 mutant alleles used in this study, *cipk9-1* and *cipk9-2*, had already been confirmed by Pandey *et al.* (2007).

### Generation of overexpressing transgenic plants

To generate Arabidopsis overexpression constructs, the complete ORF of *AP2C1* was PCR-amplified and cloned at the *BamH*I and *Sal*I restriction sites of the modified pCAMBIA1300 vector under the control of the CaMV 35S promoter. Primers used are listed in Supplementary Table S1. Overexpressing transgenic plants were generated according to Singh *et al.* (2015). Transgenic plants were confirmed for AP2C1 overexpression by qPCR (according to Singh *et al.*, 2012) and used for further analysis.

### Preparation of modified growth media with variable K^+^ levels

To perform phenotype analysis under K^+^-deficient conditions, modified Murashige and Skoog (MS) medium containing different concentrations of KCl was prepared in double-distilled deionized water. This modified media contained K^+^-free 1/20 strength of MS major salts and 1× MS minor salts. K^+^-free medium was prepared by replacing MS salts with the following (1× MS): 1650 mg l^–1^ NH_4_NO_3_, 440 mg l^–1^ CaCl_2_.2H_2_O, 370 mg l^–1^ MgSO_4_.7H_2_O, 165 mg l^–1^ (NH_4_)_2_HPO_4_, 27.8 mg l^–1^ FeSO_4_.7 H_2_O, 37.3 mg l^–1^ di-sodium EDTA, 0.7495 mg l^–1^ NaI, 6.3 mg l^–1^ H_3_BO_3_, 16.9 mg l^–1^ MnSO_4_.H_2_O, 8.6 mg l^–1^ ZnSO_4_.7H_2_O, 0.25 mg l^–1^ Na_2_Mo_4_.2H_2_O, 0.016 mg l^–1^ CuSO_4_.5H_2_O, and 0.0267 mg l^–1^ CoSO_4_.6H_2_O. For solidification of the media, 1% agarose (Sigma-Aldrich, USA) was used. Varying levels of K^+^ were achieved by adding KCl to the K^+^-free media.

### Phenotype and root elongation assays

Arabidopsis seeds from the Col-0 wild-type (WT), the *cipk9* mutant alleles (*cipk9-1* and *cipk9-2*), the *ap2c1* mutant alleles (*ap2c1-1* and *ap2c1-2*), and the *AP2C1 OX* transgenic lines were surface-sterilized as above. Approximately 30 seeds were planted on modified MS-agarose medium with different concentrations of K^+^ and incubated at 4 °C for 4 d for stratification, then transferred to a growth chamber at 22 °C under long day conditions (16 h light, 8 h dark). The plates were placed vertically on a rack. After 7 d, the root growth and fresh weight of seedlings were determined and expressed as relative values compared to the wild type at each K^+^ concentration. Experiments were repeated three times and data are presented as means (±SD).

### Estimation of K^+^ content by atomic absorption spectroscopy

Arabidopsis seeds were surface-sterilized and plated on Whatman filter paper 1. The plates were supplemented with 1/20 strength (major salts) MS media. The seeds were allowed to grow at 22 °C under long-day conditions with 200 µE m^–2^ s^–1^ light intensity and 75% humidity. After 10 d of growth, seedlings were washed 6–8 times with double-distilled Milli-Q water to remove traces of K^+^. The seedlings were then supplemented with 10 µM or 10 mM KCl solution to produce K^+^-deficient and control samples, respectively. After 14 d, the seedlings were harvested and oven-dried for 48 h at 50 °C. Then 10mg samples of dried tissue were weighed in separate 15 ml test tubes, digested with 1 ml concentrated HNO_3_, and concentrated to ~100 µl at 65 °C in an oven. The digested concentrate was re-dissolved to 10 ml using double-distilled Milli-Q water. The ion content in the samples was then analysed using an AAnalyst 400 Atomic Absorption Spectrometer (Perkin Elmer, USA). A standard curve was plotted using known standard concentrations of K^+^. The experiment was performed at least three times using three technical and biological replicates.

### K^+^-deficiency treatment and expression analysis by qPCR

Arabidopsis seeds of the different genotypes were surface-sterilized and grown in a similar way as for the K^+^-content estimation above. After 10 d of growth, seedlings were treated with K^+^-sufficient (10 mM K^+^) or K^+^-deficient (10 µM K^+^) media. After treatment for 5 d seedlings were collected and immediately frozen in liquid N_2_. Total RNA isolation and cDNA preparation was carried out according to Singh *et al.* (2015) and qPCR expression analysis was performed according to Singh *et al.* (2012). Three replicate seedlings were used for each treatment.

### Statistical analysis

All the expression, phenotypic, and quantitative experiments were carried out with three replicates and the data are presented as means (±SD). Two-tailed Student’s *t*-tests were performed to determine the statistical significance among the samples.

## Results

### AP2C1 physically interacts with CIPK9

**Fig. 1.**
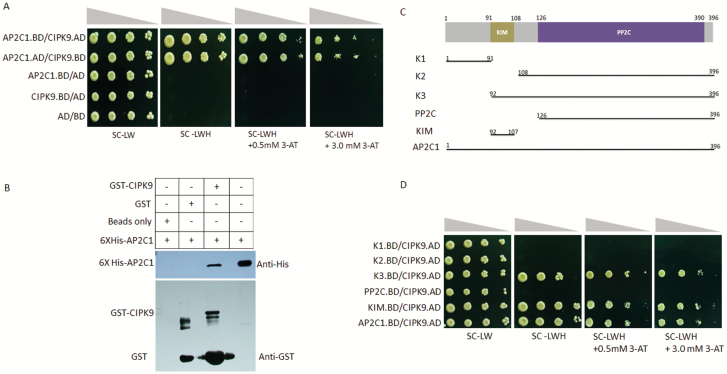
Interaction of AP2C1 with CIPK9. (A) Dilution series of yeast AH109 strains transformed with AD/BD-CIPK9 and AD/BD-AP2C1 constructs. The combination of plasmids is indicated on the left and decreasing cell densities in the dilution series are illustrated above the images. Yeast was grown on SC-LW medium, SC-LWH medium, or on SC-LWH medium containing 0.5 and 3.0 mM 3-AT. (B) *In vitro* GST pull-down assay. GST-CIPK9 and His-AP2C1 were expressed in *E. coli* and used for analysis. The western blot shows the detection of the signal using an anti-His antibody, with the anti-GST antibody as the loading control. The presence or absence of each protein in the reaction mixture is shown as + or –, respectively. (C) Scheme for making different AP2C1 deletion fragments for the interaction analysis with CIPK9. (D) Dilution series of yeast AH109 strains transformed with AD-CIPK9 and BD-AP2C1 or deletion constructs. The combination of plasmids is indicated on the left and decreasing cell densities in the dilution series are illustrated above the images. Different negative controls were used and showed no growth on the selection media.

CIPK9 has been identified as a positive regulator of K^+^-deficiency signaling in Arabidopsis (Pandey *et al.*, 2007). In order to identify the components of CIPK9-mediated signaling, a yeast two-hybrid library screening was performed and AP2C1 was identified as one of the putative interactors of CIPK9. To confirm the interaction, target-based yeast two-hybrid assays were performed. Yeast cells co-transformed with AP2C1 and CIPK9 proliferated on the selection media SC–Leu–Trp–His (SC-LWH) and on SC-LWH media containing 0.5 mM and 3.0 mM of 3-Amino-1,2,3-triazole (3-AT). Similar growth patterns were observed when the vectors were swapped (Fig. 1A). No growth was seen for the negative controls, AP2C1.BD/AD, CIPK9.BD/AD, and AD/BD, on the same medium. An *in vitro* GST pull-down assay was also performed, with *E. coli* expressing GST-CIPK9 and His-AP2C1 used for analysis. Probing with an anti-6X-His antibody showed a strong band in western blot analysis, corresponding to the size of AP2C1 when both His-AP2C1 and GST-CIPK9 were present in the reaction (Fig. 1B). The growth patterns of yeast and the GST protein pull-down assays confirmed the physical interaction between the AP2C1 and CIPK9 proteins. Moreover, interaction analysis of AP2C1 with all the Arabidopsis CIPK members (CIPK1–26), and that of CIPK9 with other PP2Cs, e.g. AIP1 and PP2CA, which are homologs of AP2C1 and previously reported in K^+^ deficiency-related functions (Lee *et al.*, 2007; Lan *et al.*, 2011), revealed that the interaction of AP2C1 with CIPK9 was specific and exclusive (see Supplementary Figs S1, S2).

### The KIM domain of AP2C1 is necessary and sufficient for interaction

AP2C1 is a MAPK phosphatase and it interacts with MAPKs through its kinase-interacting motif (KIM) at the N-terminal; mutations in this motif abolish the interaction (Schweighofer *et al.*, 2007; Umbrasaite *et al.*, 2010). To investigate whether the KIM domain was responsible for the AP2C1–CIPK9 interaction, yeast two-hybrid assays were performed (Fig. 1C, D). The growth patterns revealed that only yeast co-transformed with CIPK9 and KIM or CIPK9 and K3 (containing PP2C and KIM) grew well on the selection medium, while yeast transformed with other constructs lacking KIM could not grow. This observation established that the AP2C1-KIM domain is necessary and sufficient for the interaction with CIPK9.

### AP2C1 interacts with CIPK9 *in planta*

**Fig. 2.**
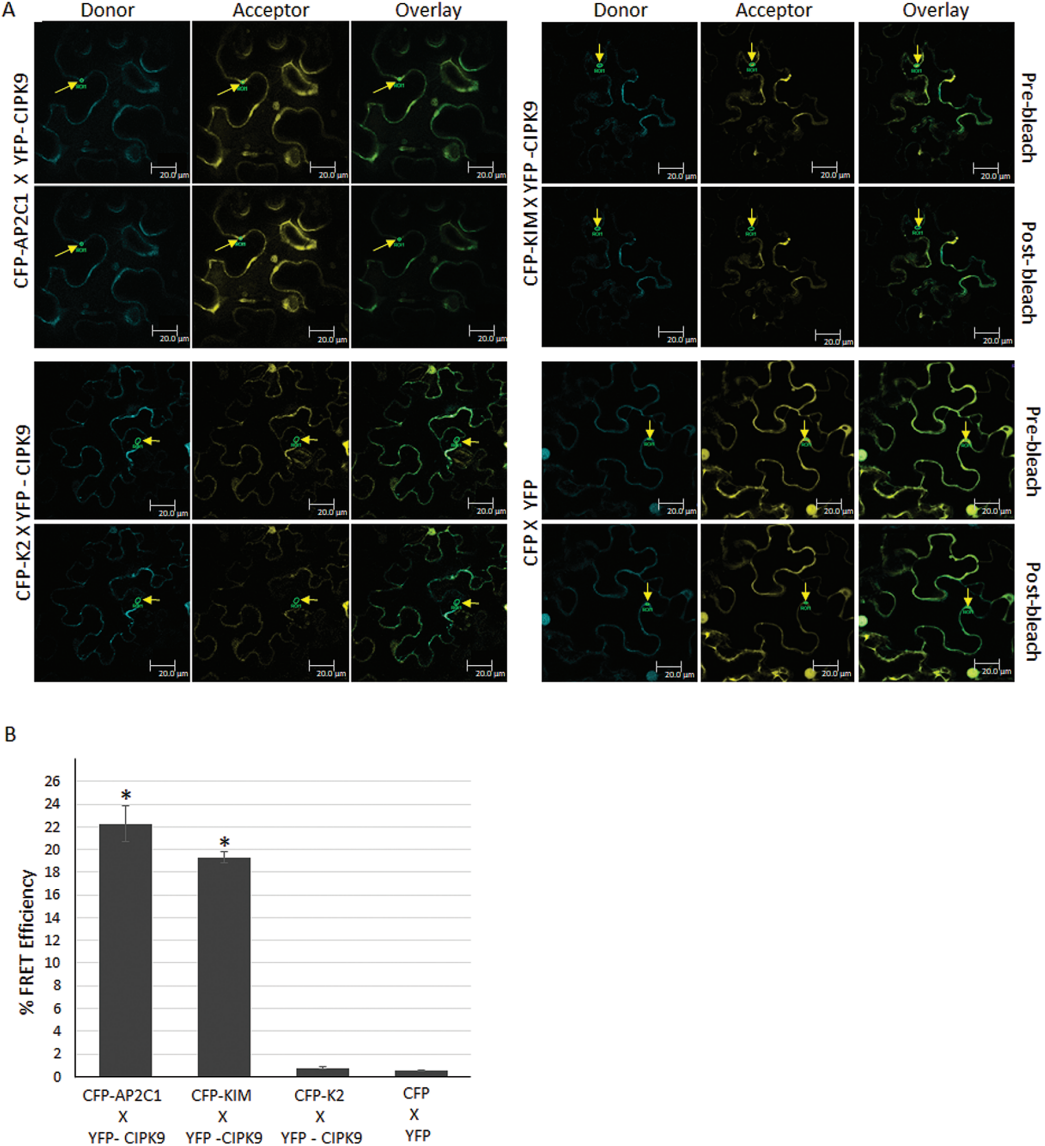
*In planta* interaction of CIPK9 and AP2C1 by fluorescence resonance energy transfer (FRET) analysis. (A) Fusion constructs of AP2C1, KIM, and K2 fragments with cyan fluorescent protein (CFP) and of CIPK9 with yellow fluorescent protein (YFP) were co-inoculated in *Nicotiana benthemiana* cells. The cells that showed expression of both CFP and YFP were targeted for FRET analysis according to the acceptor-bleaching protocol. Representative interactions are shown for different FRET combinations. Arrows indicate the region of interest selected for detailed analysis and calculation of FRET efficiency in individual cells. Scale bars are 20 µm. (B) FRET efficiency, calculated as the mean (±SD) of seven different cells. For the negative control, the efficiency was calculated for cells transformed with vectors containing CFP and YFP only (CFP X YFP). *P<0.05 compared with the negative control.

**Fig. 3.**
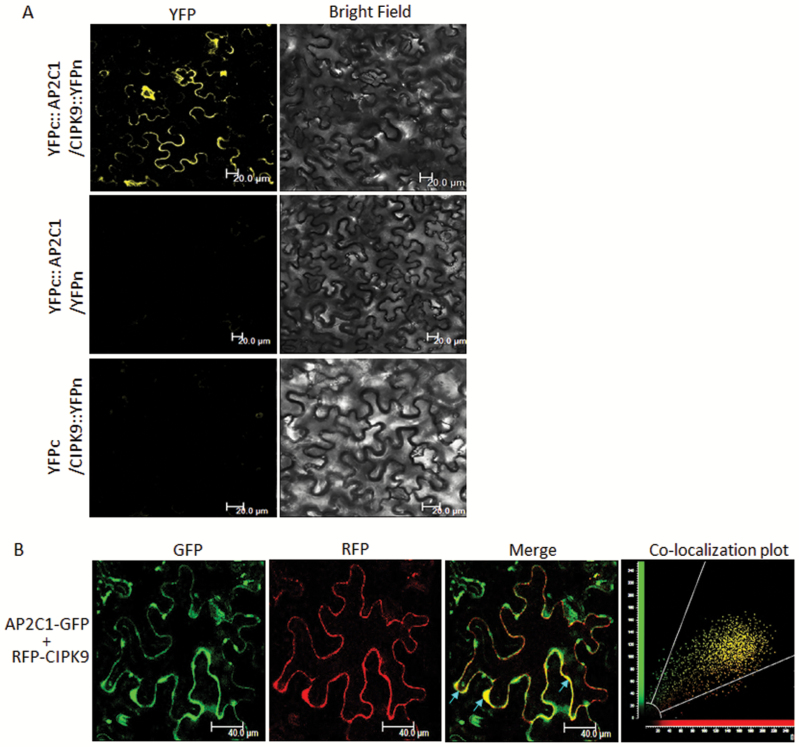
Interaction analysis of AP2C1 and CIPK9 by bimolecular fluorescence complementation (BiFC) and co-localization assays. (A) *Nicotiana benthamiana* cells co-infiltrated with YFPc::AP2C1/CIPK9::YFPn showing reconstitution of the yellow fluorescent protein (YFP) signal in the cytosol, while co-infiltration of YFPc::AP2C1/YFPn and YFPc/CIPK9::YFPn (negative controls) shows no YFP fluorescence, Scale bars are 20 µm. (B) *N. benthamiana* epidermal cells co-transformed with RFP-CIPK9 and AP2C1-GFP showing merger of the two signals in the cytosol as yellow fluorescence. The scatter-plot showing maximum yellow dots in the common region confirms the co-localization of the two proteins. Scale bars are 40 µm.

To ascertain the AP2C1–CIPK9 interaction *in planta*, FRET acceptor-bleaching analysis was performed, with CFP-AP2C1 and YFP-CIPK9 co-infiltrated into *N. bentahmaiana* cells. Cells showing the CFP/YFP signal were scanned for FRET between the two proteins. AP2C1 was found to interact with CIPK9 in the cytosolic region. Importantly, the KIM domain was also found to interact with CIPK9, whereas the K2 domain, which lacks KIM domain, did not interact with CIPK9 (Fig. 2A). Moreover, the FRET efficiency was found to be higher for CFP-AP2C1 × YFP-CIPK9 and CFP-KIM × YFP-CIPK9 than for CFP-K2 × YFP-CIPK9 and the vector control CFP × YFP, where it was almost negligible (Fig. 2B). This observation verified the interaction of AP2C1 with CIPK9 and the requirement of a KIM domain for the interaction *in planta*. In addition, to further validate the interaction BiFC and co-localization assays were performed. AP2C1 was fused to the C-terminal fragment of YFP and CIPK9 was fused to the N-terminal for the BiFC assay. Confocal microscope analysis of transformed *N. benthamiana* cells showed that the AP2C1–CIPK9 complex was formed in the cytosol, as the YFP signal was reconstituted in the cytosolic region (Fig. 3A).

For co-localization, AP2C1-GFP and RFP-CIPK9 were co-transformed in epidermal cells of *N. benthamiana*. Several cells showed strong fluorescence in both the GFP and RFP channels, and when their signals were merged a yellowish fluorescence was observed at certain locations in the cytosol (Fig. 3B). The co-localization of the two proteins was also confirmed by a scatter-plot analysis. The overlapping expression and complex-formation of AP2C1 and CIPK9 in the cytosol is indicative of their concurrent functional requirement in the cytosol.

### AP2C1 dephosphorylates the auto-phosphorylated CIPK9 *in vitro*

**Fig. 4.**
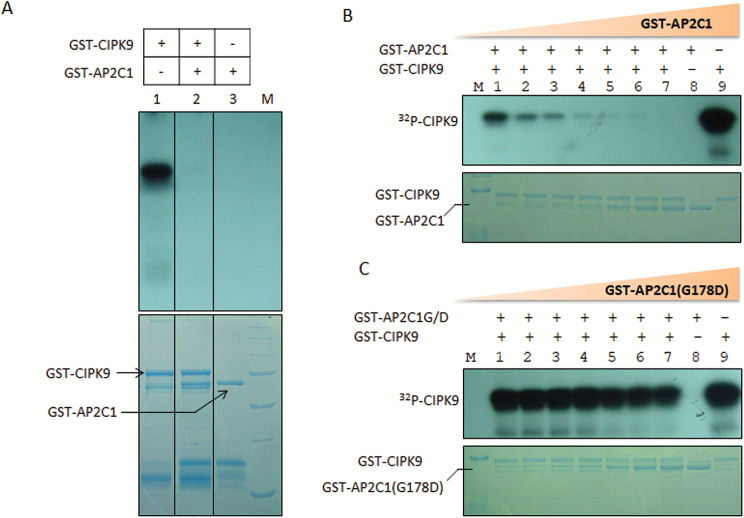
Dephosphorylation of auto-phosphorylated CIPK9 by AP2C1. (A) Autoradiogram showing detection of signals after adding different combination of GST-CIPK9 and GST- AP2C1 proteins. Lanes of interest were sliced from same autoradiogram/gel and placed together for easy representation of the data. Each sliced part is clearly demarcated by border lines. (B) To determine the dose-dependent activity of AP2C1, the concentration of GST-AP2C1 was increased gradually from 0–250 ng while GST-CIPK9 was kept constant at 500 ng in all the reactions. The autoradiogram shows the gradual decrease in the auto-phosphorylation signal of CIPK9. (C) A gradual increase in the concentration of GST-AP2C1 (G178D) from 0–250 ng did not affect the phosphorylation level of auto-phosphorylated GST-CIPK9. The presence or absence of each protein in the reaction mixture is shown as + or –, respectively.

Based on the interaction results, we hypothesized that CIPK9 might target and phosphorylate AP2C1 or that CIPK9 could be dephosphorylated by AP2C1. To test this, an *in vitro* enzymatic assay was performed. Purified GST-CIPK9 and GST-AP2C1 proteins (Supplementary Fig. S3) were used, and when ^32^P-radiolabeled GST-CIPK9 was incubated with GST-AP2C1, the level of phospholabel was reduced. Importantly, no phosphorylation was detected on AP2C1 (Fig. 4A). When the concentration of the AP2C1 protein was gradually increased while the concentration of the CIPK9 protein was kept constant, a gradual decrease in the phospholabel of CIPK9 was detected (Fig. 4B). To confirm the specificity of dephosphorylation, a mutated GST-AP2C1 protein was created, where an important glycine (G) residue at position 178 was substituted by aspartate (D) through site-directed mutagenesis. This mutation in the PP2C catalytic domain has been shown to block the phosphatase activity *in vitro* (Sheen, 1998; Meskiene *et al.*, 2003). It was observed that the mutated AP2C1 (G178D) did not dephosphorylate the auto-phosphorylated CIPK9 (Fig. 4C). These findings confirmed that CIPK9 does not phosphorylate AP2C1 *in vitro*, but that AP2C1 dephosphorylates the auto-phosphorylated CIPK9.

### **ap2c1** mutants are tolerant to low-K^+^ conditions

**Fig. 5.**
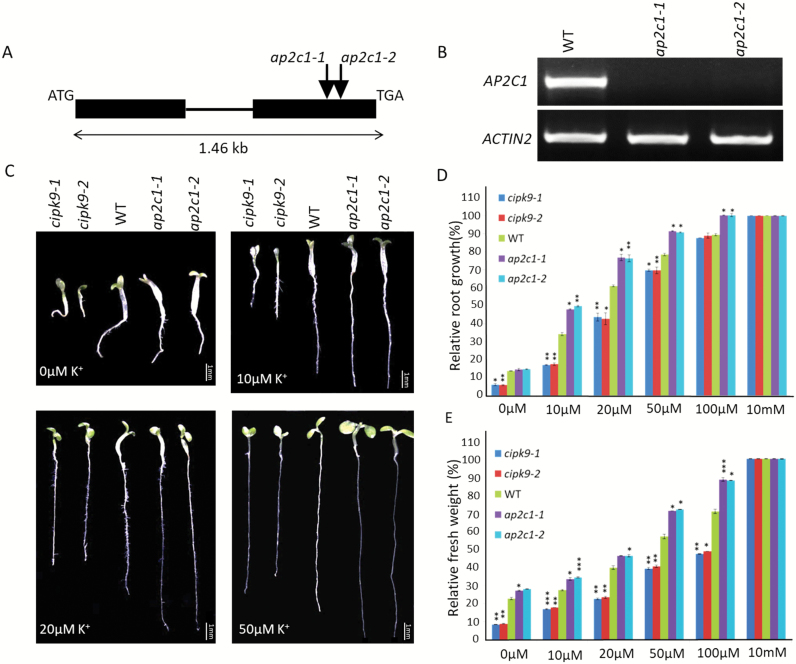
Phenotypes of seedlings of AP2C1 and CIPK9 null mutants on K^+^-deficient media. (A) Schematic of the *AP2C1* gene structure. Exons (closed boxes) and introns (lines) are indicated. The positions of the T-DNA insertions are indicated by arrows. (B) RT-PCR analysis using AP2C1 gene-specific primers confirmed the null mutant alleles of AP2C1 (*ap2c1-1*, *ap2c1-2*). Expression of *ACTIN2* was used as an endogenous control. (C) Growth of *cipk9-1*, *cipk9-2*, wild-type (WT, Col-0), *ap2c1-1*, and *ap2c1-2* under different K^+^ concentrations (as indicated) after 7 d. The 10 mM K^+^ concentration was used as the control (similar to half-strength MS medium). Scale bars are 1 mm. (D) Quantitative assessment of AP2C1 and CIPK9 null mutant phenotypes under different K^+^ conditions after 7 d. The data show root growth relative to that of the WT at 10 mM K^+^.(E) Fresh weight of 7-d-old seedlings grown with different K^+^ concentrations. The data show fresh weight relative to that of the WT at 10 mM K^+^. In (D, E) 20 seedlings of each genotype were used for analysis and three independent experiments were conducted (*n*=3); data are means (±SD). *P<0.05, ** *P*<0.01, ***P<0.005.

Based on the interaction between AP2C1 and CIPK9, and the dephosphorylation of CIPK9 by AP2C1, we hypothesized that AP2C1 might regulate CIPK9 function in low-K^+^ responses. To test this, two T-DNA insertion alleles of AP2C1 (*ap2c1-1* and *ap2c1-2*) were analysed (Fig. 5A, B). In addition, two mutant alleles of CIPK9 (*cipk9-1* and *cipk9-2*) reported earlier by Pandey *et al.* (2007) were also used in this analysis.

Phenotype assays were performed for both the mutant alleles of *ap2c1* and *cipk9* together with the Col-0 wildtype (WT) on low-K^+^ MS media. Slight differences in growth were observed for *ap2c1-1*, *ap2c1-2*, and WT at 0 μM K^+^, while both *cipk9-1* and *cipk9-2* showed strong hypersensitivity (Fig. 5C; Supplementary Fig. S4). On 10 μM K^+^ medium, visible differences could be seen in the root growth of *ap2c1-1*, *ap2c1-2*, and the WT. Both the mutant alleles of AP2C1 showed better root growth at 10, 20, and 50 μM concentrations of K^+^. At 10 mM K^+^ (equivalent to the concentration of K^+^ in 1/2 MS media) there was no difference in the seedling growth of the mutants and the WT (Fig. 5D). At most of the low-K^+^ concentrations, the *cipk9* mutant alleles consistently showed hypersensitivity; as the K^+^ concentration reached 100 μM, differences in the root growth between the *cipk9* mutants and the WT diminished. However, *ap2c1* mutant plants showed better root growth even at 100 μM K^+^. Detailed analysis of the seedlings revealed that the *ap2c1* mutants not only had better root growth but also showed better shoot growth. An examination of the seedling at 10, 20, and 50 μM K^+^ showed that both the mutant alleles of *ap2c1* had bigger and more expanded cotyledons than the WT, and even more so than both the *cipk9* mutant alleles. This observation was further supported by quantification of the relative fresh weight (Fig. 5E). These findings confirm that the *cipk9* mutant is sensitive to low-K^+^ conditions while the *ap2c1* mutant is tolerant.

### AP2C1 overexpression renders plants sensitive to low-K^+^ conditions

**Fig. 6.**
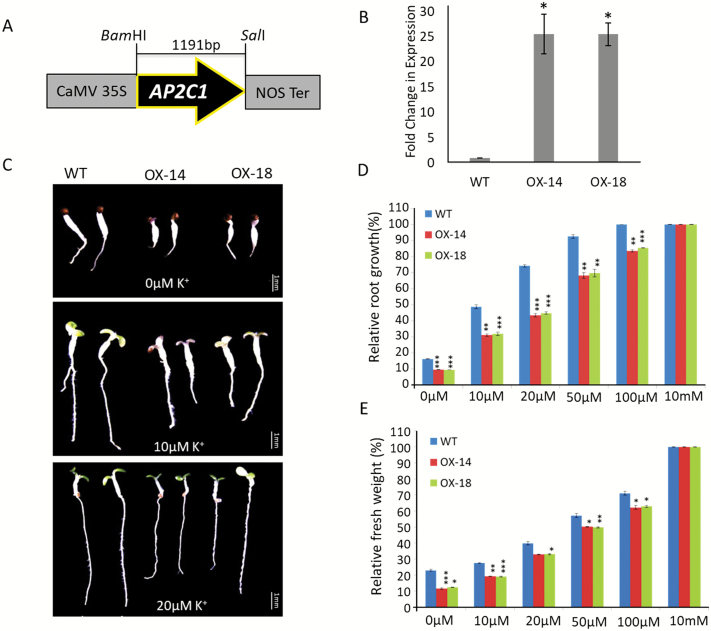
Phenotypes of transgenic AP2C1-overexpression lines on K^+^-deficient media. (A) Schematic representation of the construct used for overexpression of AP2C1 in Arabidopsis. (B) qPCR analysis of two AP2C1-overexpression lines (OX-14 and OX-18) confirming ~25-fold higher expression than the wild-type (WT, Col-0). The data are means (±SE) of three replicates. * *P*<0.05 for OX lines relative to the WT. (C) Growth of the WT, OX-14, and OX-18 on low-K^+^ concentrations after 7 d. Scale bars are 1 mm. (D) Root growth of seedlings after 7 d with different K^+^ concentrations. The data show root growth relative to that of the WT at 10 mM K^+^. (E) Fresh weight of 7-d-old seedlings grown on different K^+^ concentrations. 20 seedlings of each genotype were used for analysis in three independent experiments (*n*=3); data are means (±SD). *P<0.05, **P<0.01, ***P<0.005.

To determine the effect of ectopic expression of AP2C1 on plant growth under low-K^+^ conditions, we generated the overexpression (OX) transgenic lines OX-14 and OX-18 (Fig. 6A, B). Both lines showed a phenotype that was opposite to *ap2c1* mutants under low-K^+^ conditions and exhibited sensitivity in root growth (Fig. 6C; Supplementary Fig. S5). While no significant differences in root growth were observed between the OX lines and the WT at 10 mM K^+^, seedlings of both the lines showed reduced root growth at 0 to 100 μM K^+^. Quantitative analysis supported the growth phenotype, and the maximum difference in root growth was recorded at 20 μM K^+^, where the WT showed ~75% growth relative to the control (10 mM K^+^) and both the OX lines showed less than 45% (Fig. 6D). The OX-14 and OX-18 seedlings had reduced fresh weight relative to the WT at micromolar K^+^ concentrations, whilst there were no significant differences at 10 mM K^+^ (Fig. 6E).

### K^+^ homeostasis and related gene expression support the tolerance of the **ap2c1** mutant to low-K^+^ conditions

**Fig. 7.**
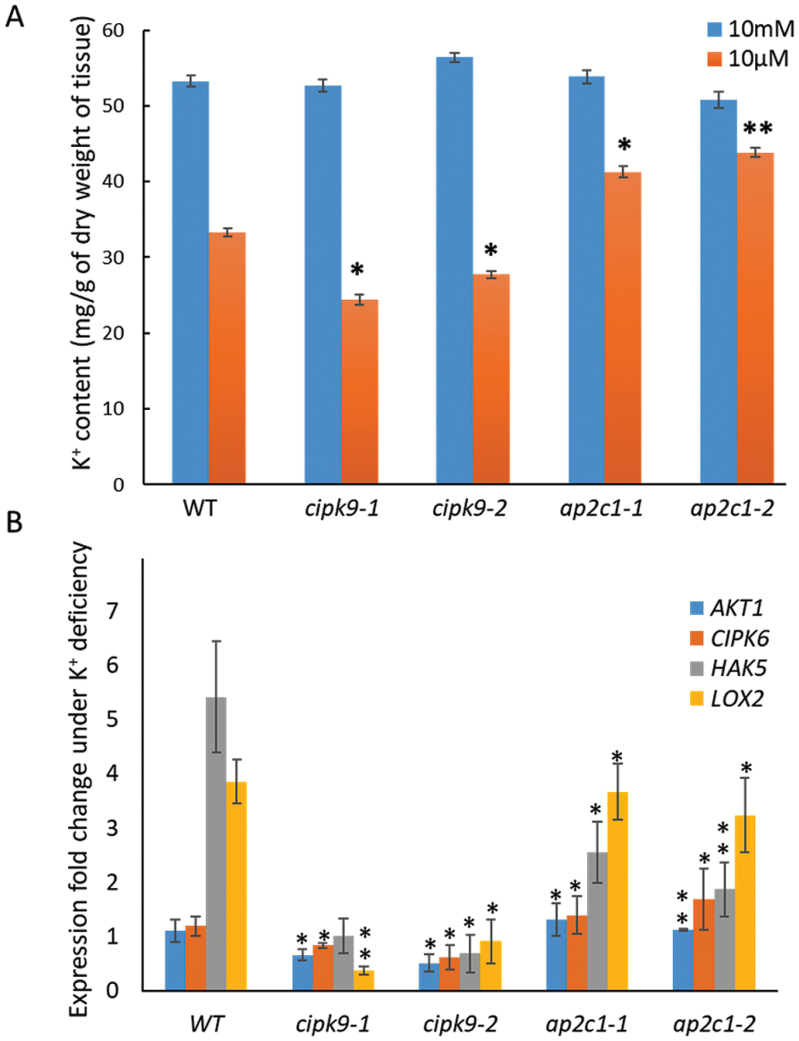
K^+^ ion content and K^+^ deficiency-related gene expression in *cipk9* and *ap2c1* mutants. (A) K^+^ content was determined in the wild-type (WT), *ap2c1-1*, *ap2c1-2*, *cipk9-1*, and *cipk9-2* under K^+^-sufficient (10 mM K^+^) and K^+^-deficient (10 µM K^+^) conditions by atomic absorption spectrophotometry. The experiment was repeated three times with three biological replicates for each sample. *P<0.05; **P<0.01. (B) Relative expression of *AKT1*, *CIPK6*, *HAK5*, and *LOX2* in the WT, *ap2c1-1*, *ap2c1-2*, *cipk9-1*, and *cipk9-2* under K^+^-deficient conditions with respect to K^+^-sufficient conditions. Expression was analysed by qPCR and relative expression was calculated by the 2^–ΔΔ*C*t^ method (Livak and Schmittgen, 2001) using three replicates for each sample. Values are means (±SE). *P<0.05, **P<0.01 compared with the WT.

To understand the possible mechanism of the tolerance of the AP2C1 mutant under low-K^+^ conditions and the functional relationship with CIPK9, the total K^+^ content was estimated under low- and sufficient-K^+^ growth conditions. Under sufficient conditions (10 mM K^+^) the WT, *cipk9-1*, *cipk9-2*, *ap2c1-1*, and *ap2c1-2* seedlings contained almost similar amounts of K^+^ (55 mg g–1 dry weight, Fig. 7A). Under deficient conditions (10 µM K^+^), whilst the WT contained approximately 32mg g^–1^ K^+^, both the mutant alleles of *cipk9* had reduced K^+^ content (23–26 mg g^–1^). In contrast, both *ap2c1-1* and *ap2c1-2* had higher K^+^ contents than the WT (41–44 mg g^–1^). Various transporters/channels and enzymes are known to be involved in K^+^ uptake, transportation, and homeostasis. Therefore, to understand the mechanisms underlying the variable levels of K^+^ content in the *cipk9* and *ap2c1* mutants, expression analysis was carried out for some of the K^+^ transport- and homeostasis-related genes, namely *AKT1*, *CIPK6*, *HAK5*, and *LOX2* (Armengaud *et al.*, 2004). Under low-K^+^ conditions, no significant differences were observed in the expression of *AKT1* and *CIPK6* in the WT, whereas, *HAK5* and *LOX2* had significantly higher expression levels (Fig. 7B). Importantly, expression of all the genes was lower in both *cipk9-1* and *cipk9-2*. Expression of *AKT1* and *CIPK6* in both *ap2c1-1* and *ap2c1-2* was higher than in the *cipk9* mutants and was comparable to the WT. Notably, the expression levels of *HAK5* and *LOX2* in *ap2c-1* and *ap2c1-2* were much higher than in the *cipk9* mutants and almost approached the levels observed in the WT. These findings suggest a possible regulatory role of these and similar K^+^ homeostasis-related genes in the uptake and distribution of K^+^ in the *ap2c1* and *cipk9* mutants.

## Discussion

In plants, the Ca^2+^ signal generated by K^+^ deficiency is primarily decoded by the CBL-CIPK complex and transduced downstream by CIPK-mediated phosphorylation of target proteins (Luan *et al.*, 2009). Specific CBL-CIPK complexes such as CBL1/9-CIPK23 and CBL4-CIPK6 regulate K^+^ uptake and distribution in plants through activation of the Shaker channels AKT1 and AKT2, respectively (Xu *et al.*, 2006; Held *et al.*, 2011). Ca^2+^-sensing kinases, CDPKs, are also implicated in the regulation of the activity of K^+^ ion channel (Simeunovic *et al.*, 2016; Singh *et al.*, 2017). CPK13 phosphorylates two Shaker channel subunits, KAT1 and KAT2, in the guard cells to control stomatal closure (Ronzier *et al.*, 2014). Recently, a functional screen in *Xenopus* oocytes showed that CPK33 acting in a Ca^2+^-dependent manner stimulated the activity of the outward-rectifying voltage-gated K^+^ Shaker channel GORK, and thus CPK33 promotes Ca^2+^-dependent stomatal closure (Corratgé-Faillie *et al.*, 2017). Interestingly, in addition to K^+^ uptake, the CBL1/CBL9–CIPK23 module has also been shown to be involved in stomatal regulation under dehydrating conditions (Cheong *et al.*, 2007). Therefore, involvement of CDPKs in K^+^ uptake and distribution is quite possible as drought stress and K^+^ deficiency are interlinked processes in plants (Weinl and Kudla, 2009).

Like CIPK23, CIPK9 is known to be involved in regulation of low-K^+^ signaling in Arabidopsis (Pandey *et al.*, 2007). It has been identified as a positive regulator of low-K^+^ signaling because its mutants are hypersensitive to low-K^+^ conditions. In contrast, another study showed that CIPK9 might be a negative regulator because it was found that *cipk9* mutant plants were more tolerant to low-K^+^ conditions (Liu *et al.*, 2013). These contradictory observations could be accounted by the differences in the experimental procedures between the studies, including differing growth media and growth conditions, the differing phenotypes observed, and the timing of phenotyping. In the CBL1/CBL9–CIPK23–AKT1 signaling module, the AKT1 channel is phosphorylated by CIPK23, and AIP1 (PP2C) dephosphorylates it, thereby reversing the AKT1 channel K^+^-uptake activity (Lee *et al.*, 2007). In search of upstream and downstream components of CIPK9-mediated K^+^-deficiency signaling, we identified another PP2C, designated as AP2C1, which interacts with CIPK9 *in vitro* and *in planta*. Previously, AP2C1 was recognized as a MAPK phosphatase and its KIM motif was found to be responsible for interactions with MAPK4 and MAPK6 (Schweighofer *et al.*, 2007). Interestingly, mapping of different regions of AP2C1 showed that the KIM domain of AP2C1 is necessary and sufficient for its interaction with CIPK9 (Fig. 1C, D). Thus, the KIM motif could be a conserved structural feature of AP2C1, which might facilitate its interaction with different classes of kinases. More importantly, it might be crucial in conferring functional specificity to AP2C1 by governing interactions with different proteins.

*In planta* interaction analyses for CIPK9 and AP2C1 were carried out in *N. benthamiana* leaves because higher expression of CIPK9 has been observed in leaves than in roots (Pandey *et al.*, 2007; Liu *et al.*, 2013). In addition, AP2C1 has been shown to interact with MAPKs in leaf protoplasts (Schweighofer *et al.*, 2007). Our FRET, BiFC, and co-localization analyses revealed that AP2C1 interacts with CIPK9 in the cytosol, in close proximity to the membrane (Figs 2, 3). It could be assumed that in the cytosol CIPK9 might target and regulate a K^+^ ion channel/transporter located at the membrane (either the plasma membrane or endomembrane). It has been postulated that CIPKs in general are cytoplasmic proteins and that their final cellular destination is influenced by interactions with other protein partners, mainly CBLs (Waadt *et al.*, 2008; Batistič *et al.*, 2010). Channels can also facilitate translocation of CIPKs to the membrane even in the absence of CBLs, for example AKT1 with CIPK23, and AKT2 with CIPK6 (Xu *et al.*, 2006; Held *et al.*, 2011). Previously, it has been shown that CBL2 and CBL3 mediate the targeting of CIPK9 to the tonoplast membrane (Liu *et al.*, 2013; Tang *et al.*, 2015). The target of CIPK9 at the tonoplast is not known but it could be assumed that CIPK9 may phosphorylate and regulate the crucial two-pore K^+^ (TPK1) channel (located at tonoplast membrane), leading to K^+^ efflux from the vacuole to cytosol, thereby contributing to the maintenance of K^+^ homeostasis. Recently, CIPK9 was shown to interact with and phosphorylate a plasma membrane-localized Ca^2+^ pump, ACA8, in Arabidopsis (Costa *et al.*, 2017). This interaction and phosphorylation event involving ACA8-CIPK9 led to changes in the stimulus-induced dynamics of cytosolic Ca^2+^. Therefore, it could be hypothesized that other transporters/channels such as cation/Ca^2+^ exchangers (CCX), which are involved in cross-membrane transportation of cytosolic Ca^2+^ against its electrochemical gradient, could also be targeted by CIPK9 to fine-tune the Ca^2+^ level in the cytosol and to regulate K^+^ uptake. Another mode of regulation of K^+^ uptake could be transcriptional control of ion channels/transporters through CIPK9. The gene encoding *AtHAK5* is known to be readily induced under low-K^+^ conditions (Gierth *et al.*, 2005). Recently, the transcription factor AtARF2 was shown to regulate the expression of *AtHAK5* (Zhao *et al.*, 2016). Under normal conditions, AtARF2 binds to the *AtHAK5* promoter and inhibits its expression, whereas under low-K^+^ conditions AtARF2 is rapidly phosphorylated and detached from the promoter, and hence *AtHAK5* expression is reinstated. Therefore, CIPK9 might phosphorylate a similar transcription factor to enhance the transcription of key K^+^ channels such as HAK5 under low-K^+^ conditions, provided that an interacting partner such as CBL translocates CIPK9 to nucleus. However, we did not find CIPK9 or its complex with AP2C1 in the nucleus, and hence the possibility of this mode of regulation appears remote and requires further experimental investigation.

Several plant PP2Cs have been shown to regulate diverse protein kinase pathways in stress- and development-related processes (Meskiene *et al.*, 2003; Kuhn *et al.*, 2006; Schweighofer *et al.*, 2007). Importantly, multiple CIPKs have been found to interact with PP2Cs, such as ABA insensitive 1 (ABI1) and ABI2 (Ohta *et al.*, 2003). However, in such interactions it is unclear whether CIPK phosphorylates PP2C or PP2C dephosphorylates CIPK *in vivo*. Alternatively, a CIPK-PP2C complex might form a kinase/phosphatase signaling module and modulate the phosphorylation status of the target proteins (Weinl and Kudla, 2009). Therefore, in order to decipher the role of the CIPK9-AP2C1 interaction and the possible mode of regulation by these two molecules, we performed *in vitro* enzyme activity assays. These assays revealed that AP2C1 dephosphorylates auto-phosphorylated CIPK9, while CIPK9 does not phosphorylate AP2C1 (Fig. 4). Thus, dephosphorylation by AP2C1 could be the regulatory mechanism for CIPK9 function; however, *in vivo* confirmation of this mechanism is still required.

To determine the functional relevance of CIPK9 regulation by AP2C1, we adopted a genetic approach. Phenotype analysis on low-K^+^ media showed that both the *ap2c1* mutant alleles (*ap2c1-1* and *ap2c1-2*) were tolerant to K^+^-deficient conditions. Whereas the *cipk9* mutants (*cipk9-1* and *cipk9-2*) were hypersensitive in early root growth and seedling development under K^+^-deficient conditions (Fig. 5). In contrast, AP2C1-overexpressing lines showed sensitivity to low-K^+^ conditions; however, the sensitivity was not as strong as in the *cipk9* mutants (Fig. 6). To determine the tolerance/susceptibility mechanisms of AP2C1 and CIPK9 null mutants and the possible functional relationship between them, the dynamics of total K^+^ content were analysed. The higher K^+^ content of *ap2c1* mutants seedlings (Fig. 7A) suggested that they were able to take up greater amounts of K^+^ from the growth environment under deficient conditions and efficiently transport it to different parts, whereas the *cipk9* mutants were less efficient in K^+^ uptake and transportation. This implied that some K^+^ uptake-, transport-, and homeostasis-related components may be regulated by the CIPK9 signaling pathway and hence they are expressed at higher levels in the *ap2c1* mutants due to removal of AP2C1 inhibition of CIPK9 activity. In order to test this hypothesis, expression analysis was undertaken for some of the crucial K^+^ uptake- and homeostasis-related genes, namely *AKT1*, *CIPK6*, *HAK5*, and *LOX2*. Down-regulation of these genes in the *cipk9* mutants and relatively higher expression of most of them in the *ap2c1* mutants supported the variable levels of K^+^ content that were observed (Fig. 7B). These findings suggest that *HAK5* and *LOX2* might enable the *ap2c1* mutants to take up and retain higher amounts of K^+^, which can then be utilized in vital cellular processes under K^+^-deficient conditions. Importantly, the contrasting expression patterns of these genes and the lower K^+^ content in the *cipk9* mutants suggest that a functional relationship exists between AP2C1 and CIPK9 under low-K^+^ growth conditions. These K^+^ uptake- and homeostasis-related components could be the real targets of the CIPK9-AP2C1 modules; however, further experimental evidence is required to verify this hypothesis. These findings also indicate that unlike CIPK23, CIPK9 may not target the crucial K^+^ channel AKT1, as expression of AKT1 was not significantly altered in the *cipk9* or *ap2c1* mutants. In fact, Pandey *et al.* (2007) examined the relationships of CIPK9 with AKT1 and some other transporters but could not observe any interactions. Furthermore, we performed an interaction analysis of AKT1 with AP2C1 using a yeast two-hybrid assay but found that, unlike AIP1, AP2C1 did not interact with AKT1 (data not shown). Thus, CIPK9-AP2C1 possibly constitutes a separate signaling cascade for the low-K^+^ response in Arabidopsis.

In conclusion, this study demonstrates that the interaction between AP2C1 and CIPK9, with dephosphorylation of CIPK9 by AP2C1, regulates the signaling and responses under K^+^ deficiency in Arabidopsis. This CIPK9–AP2C1 module could be an alternate signaling pathway to the already established CIPK23–AKT1–AIP1 module, or it might function in parallel to improve K^+^ uptake and/or homeostasis under low-K^+^ conditions.

## Supplementary data

Supplementary data are available at *JXB* online.

Table S1. List of primers used for preparation of constructs and qPCR analyses

Fig. S1. Interaction analysis of AP2C1 with the entire Arabidopsis CIPK family.

Fig. S2. Interaction analysis of CIPK9 with AP2C1 homologs.

Fig. S3. AP2C1 and CIPK9 protein expression in *E. coli*.

Fig. S4. Phenotype analysis of AP2C1 and CIPK9 null mutants on K^+^-deficient media.

Fig. S5. Phenotype analysis of transgenic AP2C1-overexpressing lines on K^+^-deficient media.

Supplementary Table S1

Supplementary Figures
