# Supplementary material for: A protein phosphatase 2C, AP2C1, interacts with and negatively regulates the function of CIPK9 under potassium-deficient conditions in Arabidopsis
Source: J Exp Bot. 2018 May 15;69(16):4003–15. doi: 10.1093/jxb/ery182 (PMC6054203; doi:10.1093/jxb/ery182)
Supplement: Supplementary Table S1 [file ery182_suppl_supplementary_table.pdf]

Table S1: List of primers used for various constructs preparations and qPCR analyses

Purpose	Vector	Primer	Sequence
Y2H/Localization	pGAD/pGBT/GFP	AP2C1F BamHI	5'TGGATCCTATGTCTTGCTCCGTCGCC3,
Protein Expression	pGEX4T-1/pET28a	AP2C1F BamHI	5'AGGATCCATGTCTTGCTCCGTCGCC3'
Arabidopsis OX	pGKP479	AP2C1F BamHI	5'TGGATCCATGTCTTGCTCCGTCGCC3'
BiFC, FRET	p-ENTR/DTOPO, pSPYCE-35S	AP2C1-TOPO-F	5'CACCATGTCTTGCTCCGTCGCC3'
BiFC, FRET	p-ENTR/DTOPO, pSPYCE-35S	AP2C1-TOPO-R	5'TATGAACTGGCGTAAAGGG3'
Y2H/Protein expression	pGAD/pGBT/pGEX4T-1/pET28a	AP2C1R SalI	5'CGTCGACCTATATGAACTGGCGTAAAGGG3'
Arabidopsis OX	pGKP479	AP2C1R SalI	5'CGTCGACCTATATGAACTGGCGTAAAGGG3'
Localization	GFP	AP2C1R SalI	5'CGTCGACGAACTGGCGTAAAGGGATC3'
Y2H	pGAD/pGBT	K1F-BamHI	5'TGGATCCTATGTCTTGCTCCGTCGCC3'
Y2H	pGAD/pGBT	K1R-SalI	5'AGTCGACCGGAGGAGAGGATGCAGA3'
Y2H	pGAD/pGBT	K2F-BamHI	5'AGGATCCAGGTGTTGCTGGATTTGTAGC3'
Y2H	pGAD/pGBT	K2/3/PP2Cc R-SalI	5'CGTCGACCTATATGAACTGGCGTAAAGGG3'
Y2H	pGAD/pGBT	K3F-BamHI	5'AGGATCCAGGAGGTGTTTTAAAGAGGA'
Y2H	pGAD/pGBT	PP2CcF-BamHI	5'AGGATCCAAGAGAGGAGTGTAGAGAG3'
Y2H	pGAD/pGBT	KIM-F-BamHI	5'TGGATCCAGGAGGTGTTTTAAAGAGG3'
Y2H	pGAD/pGBT	KIM-R-SalI	5'AGTCGACAATCGGTATATCAAGCC3'
qPCR		AP2C1 F-RT	5'GATCACCGCCCGTCTAGAGA3'
qPCR		AP2C1 R-RT	5'CCGTGAAACGTATCAACATATCCT3'
qPCR		AKT1 F	5'ATTCCCTCAGAGGCTGCTATGA3'
qPCR		AKT1 R	5'ACGCCAAAGACGAAGCATGT3'
qPCR		CIPK6 F	5'CGGAGTATCGCCGGTGAA3'
qPCR		CIPK6 R	5'CGTGTTTCCGGTGGCAAT3'
qPCR		HAK5 F	5'CATCACGAGCACTTCATGTCTG3'
qPCR		HAK5 R	5'CTCTGTTTGATCCTCCACGGA3'
qPCR		LOX2 F	5'GCATCCTCATTTCCGCTACAC3'
qPCR		LOX2 R	5'CCACCTCCGTTGACAAGACTTT3'
