# Supplementary material for: A protein phosphatase 2C, AP2C1, interacts with and negatively regulates the function of CIPK9 under potassium-deficient conditions in Arabidopsis
Source: J Exp Bot. 2018 May 15;69(16):4003–15. doi: 10.1093/jxb/ery182 (PMC6054203; doi:10.1093/jxb/ery182)
Supplement: Supplementary Figures [file ery182_suppl_supplementary_figures.pdf]

Fig. S1

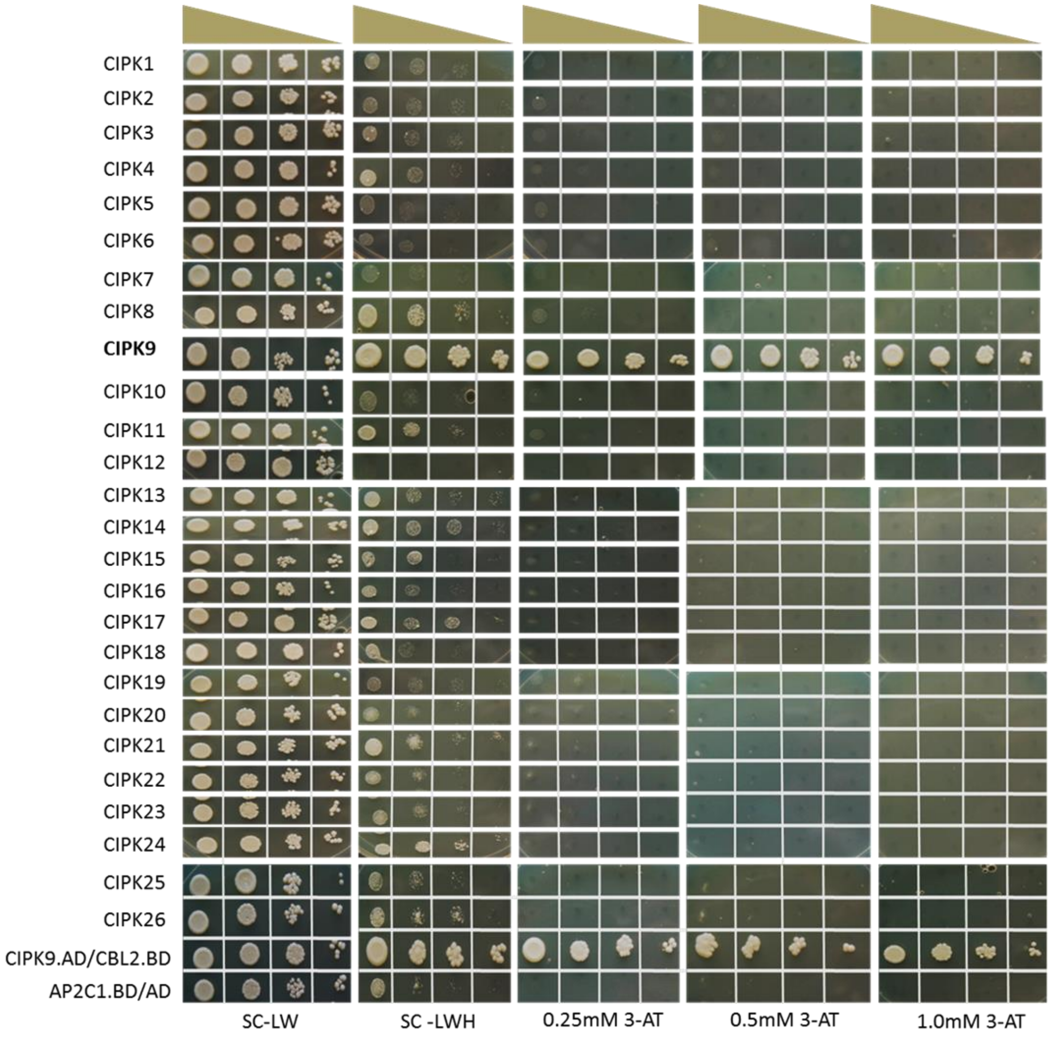

Fig. S1: Interaction analysis of AP2C1 with entire *Arabidopsis* CIPK family. AP2C1.BD and CIPKs. AD (indicated on left) plasmid constructs were used to co-transform yeast AH109. Decreasing cell densities in the dilution series are illustrated by narrowing triangles. Yeast was grown on SC-LW medium, SC-LWH medium or on SC-LWH medium containing 0.25, 0.5 and 1.0 mM 3-AT. CIPK9.AD/CBL2.BD and AP2C1.BD/AD are used as positive and negative controls, respectively.

Fig. S2

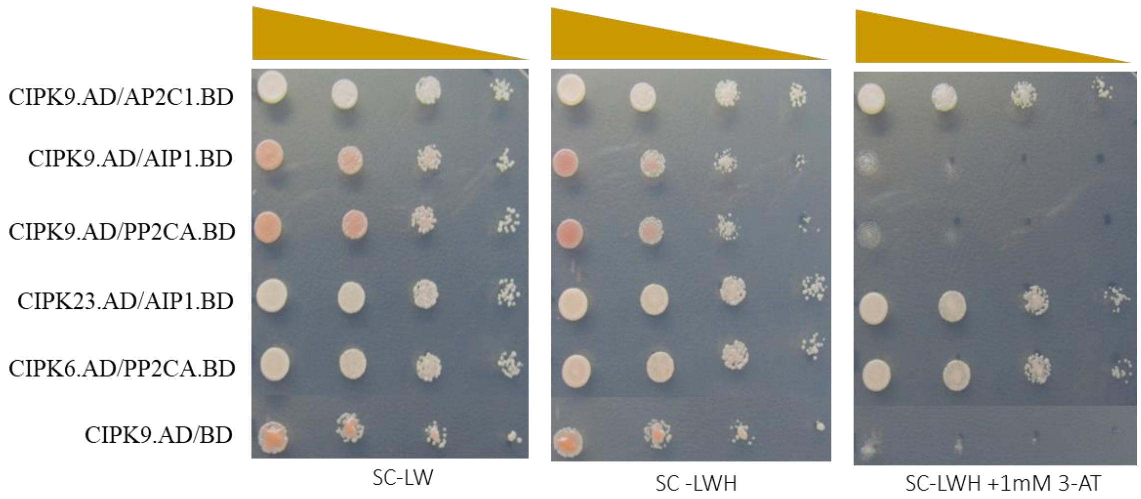

Fig. S2: Interaction analysis of CIPK9 with AP2C1 homologs. CIPK9.AD and PP2Cs.BD (indicated on left) plasmid constructs were used to co-transform yeast AH109. Decreasing cell densities in the dilution series are illustrated by narrowing triangles. Yeast was grown on SC-LW medium, SC-LWH medium or on SC-LWH medium containing 1.0 mM 3-AT. CIPK23.AD/AIP1.BD and CIPK6.AD/PP2CA.BD are used as positive controls and CIPK9.AD/BD as negative controls.

Fig. S3

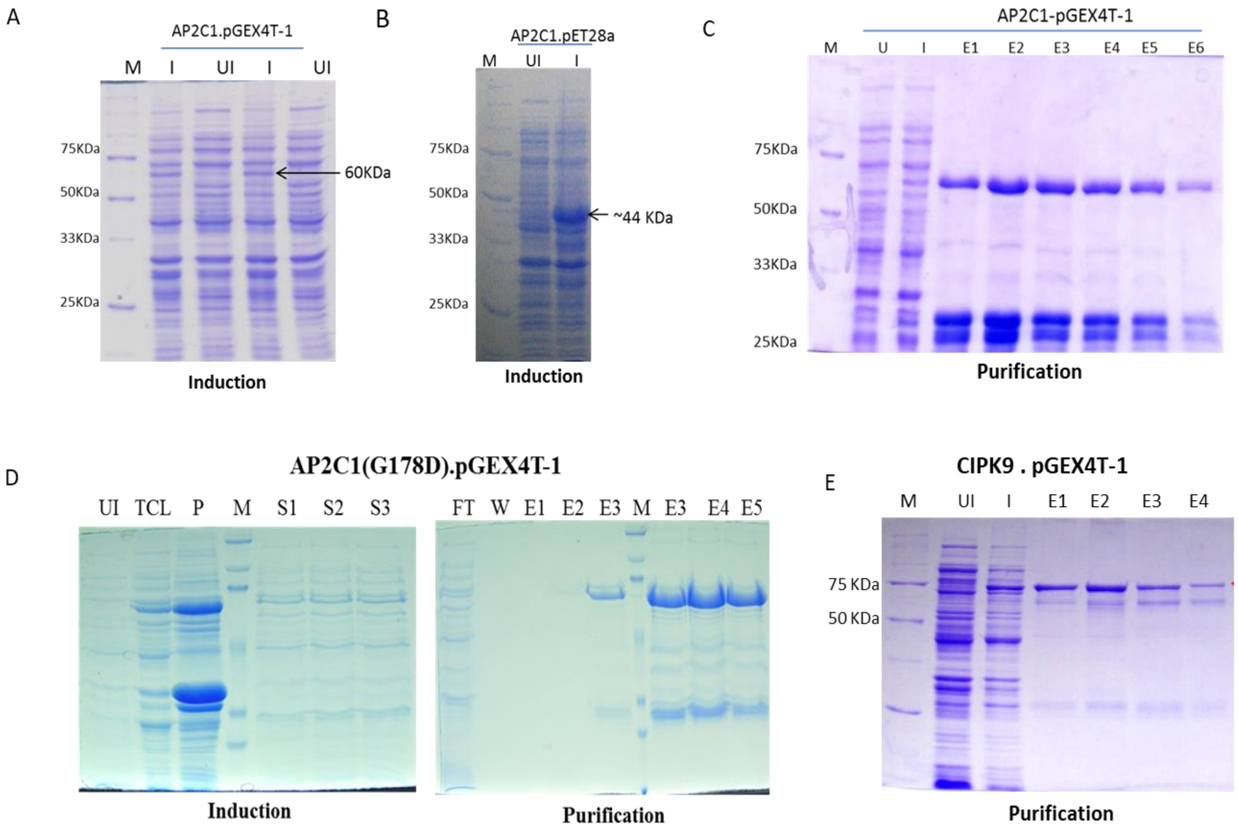

Fig. S3: AP2C1 and CIPK9 protein expression in *E. coli*. (A) SDS-PAGE gel showing induction of AP2C1 protein in fusion with GST tag at ~ 60kDa size (B) and in fusion with 6X His tag at ~ 44 kDa. (C) AP2C1 and (D) AP2C1 (G178D) protein induced in fusion with GST tag (in pGEX4T-1 vector) was partially purified through GST affinity column. SDS-PAGE gel showing desired size (~60 kDa) band in different eluted sample (E1-E6). (E) Partial purification of CIPK9 with GST fusion. M-marker, U- un-induced, I-induced samples, TLC- total cell lysate, P- pellet, S1-3 – Soluble fractions.

Fig. S4

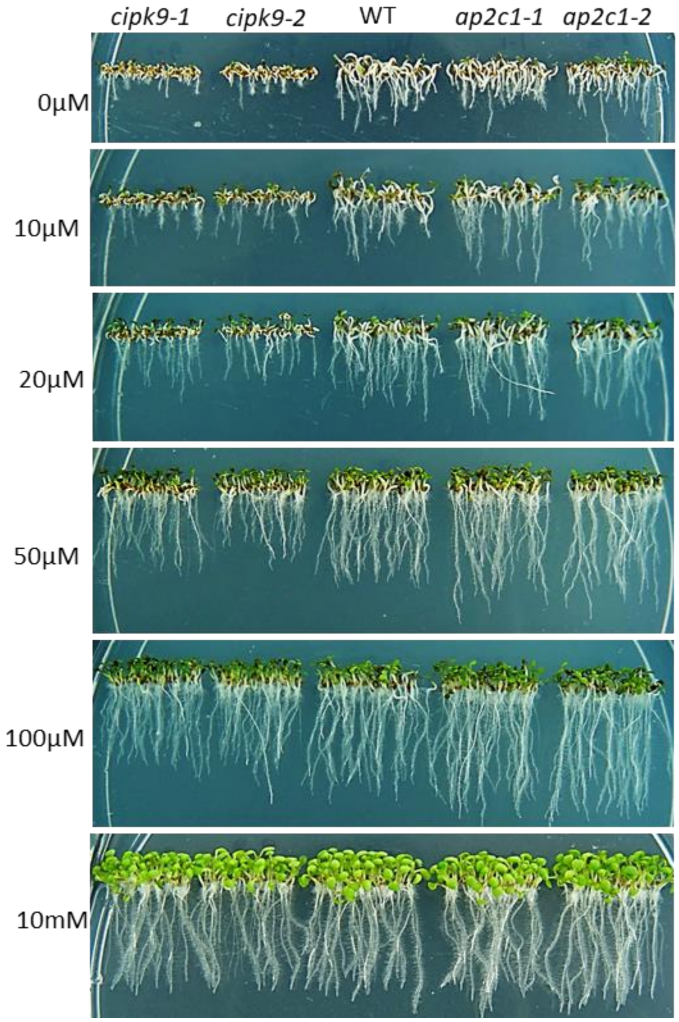

Fig. S4: Phenotype analysis of AP2C1 and CIPK9 null mutants on K^+ deficient media. Germination and vertical growth of *cipk9-1*,*cipk9-2*,WT (Col-0), *ap2c1-1* and *ap2c1-2* on low K^+ concentrations (0µM, 10µM, 20µM, 50µM, 100µM) after 7 days. 10mM K^+ concentration is used as control (similar to 1/2 MS).

Fig. S5

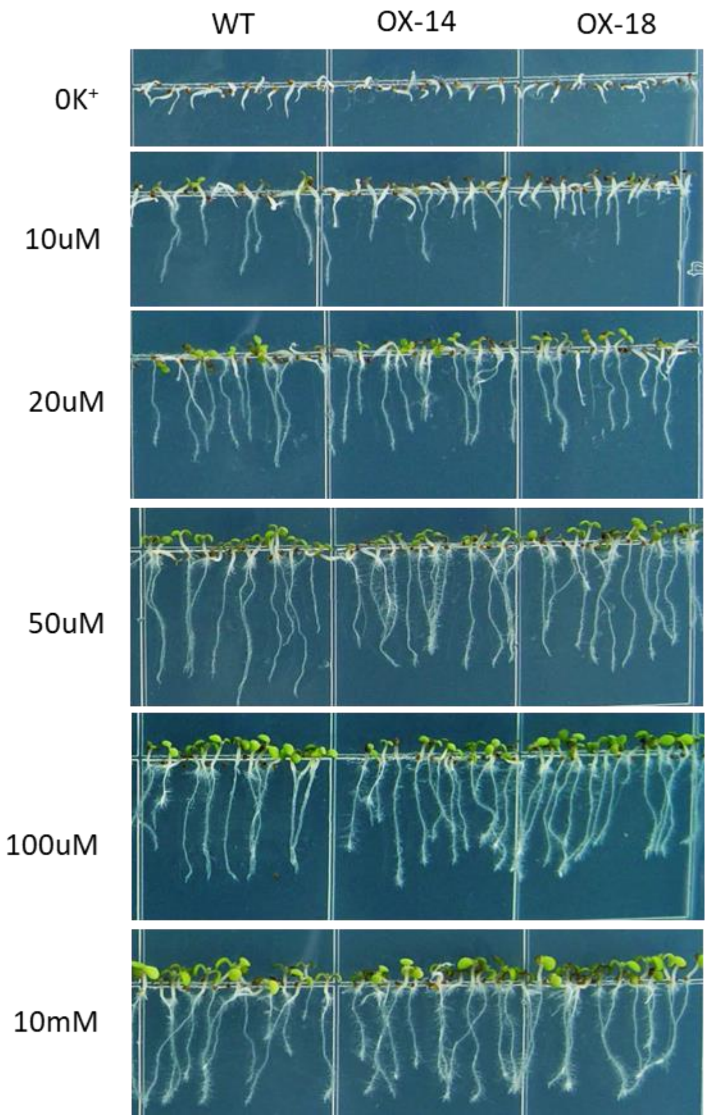

Fig. S5: Phenotypic analysis of AP2C1 overexpression transgenic on K^+ deficient media. Germination and vertical growth of WT (Col-0) and AP2C1 OX lines OX-14 and OX-18 on low K^+ concentrations (0µM, 10µM, 20µM, 50µM, 100µM) after 7 days. 10mM K^+ concentration is used as control (similar to 1/2 MS).
